# Effective access to health care in Mexico

**DOI:** 10.1186/1472-6963-14-186

**Published:** 2014-04-23

**Authors:** Juan Pablo Gutiérrez, Sebastián García-Saisó, Germán Fajardo Dolci, Mauricio Hernández Ávila

**Affiliations:** 1 Center for Evaluation Research & Surveys, National Institute of Public Health (INSP), Cuernavaca, Mexico; 2 Ministryof Health, Ciudad de Mexico, Mexico; 3 The Mexican Institute of Social Security (IMSS), Ciudad de Mexico, Mexico; 4 National Institute of Public Health (INSP), Cuernavaca, Mexico

**Keywords:** Effective access, Universal health coverage, Mexico

## Abstract

**Background:**

Effective access measures are intended to reflect progress toward universal health coverage. This study proposes an operative approach to measuring effective access: in addition to the lack of financial protection, the willingness to make out-of-pocket payments for health care signifies a lack of effective access to pre-paid services.

**Methods:**

Using data from a nationally representative health survey in Mexico, effective access at the individual level was determined by combining financial protection and effective utilization of pre-paid health services as required. The measure of effective access was estimated overall, by sex, by socioeconomic level, and by federal state for 2006 and 2012.

**Results:**

In 2012, 48.49% of the Mexican population had no effective access to health services. Though this represents an improvement since 2006, when 65.9% lacked effective access, it still constitutes a major challenge for the health system. Effective access in Mexico presents significant heterogeneity in terms of federal state and socioeconomic level.

**Conclusions:**

Measuring effective access will contribute to better target strategies toward universal health coverage. The analysis presented here highlights a need to improve quality, availability, and opportuneness (location and time) of health services provision in Mexico.

## Background

Just over a decade ago, the challenges faced by the Mexican National Health System, despite its previous accomplishments, were still significant. In 2000, almost 60% of the population in Mexico did not have access to any type of health insurance. Total health expenditure was low, which favored federal states the growth of health-care facilities with better infrastructure and higher-income individuals. This, of course, generated significant inequity in access to health services for all those people without social security coverage [1]. In that situation, individuals had to make out-of-pocket payments for all expenses related to medical care—even in public institutions with restricted funding; that led to a significant, complex economic imbalance among Mexican households. By 2000, it has been estimated that approximately two and a half million families were pushed annually to impoverishment as a result of health expenses [2].

With time, the inequity grew between the insured population and people lacking social security. In this regard, government spending in 2002 for insured individuals was 2.3-fold greater than for the uninsured sector [3]. Consequently, it became necessary to extend public health insurance schemes in Mexico. To this end, social protection in the form of the Social Health Protection System (SPSS—Sistema de Protección Social en Salud [in Spanish]) became incorporated within the national health system. The SPSS was designed to facilitate access to health services and decrease the chance of impoverishment caused by out-of-pocket expenses, particularly in the case of catastrophic health expenditure.

Overall, great progress has been made in reducing the inequality gap with health insurance. Mexico has now received international recognition for its national health system [4]. In the 10 years since its launch, the SPSS (known as *Seguro Popular*) has clearly managed to secure financing for a package of health services for a large proportion of the population. However, major challenges remain in the provision of services, which in conjunction with the persistence of some financial barriers result in a lack of effective access to health services for a significant number of people.

The consolidation of health systems depends directly on the generation and use of information and knowledge as an ongoing process [5]. Thus, it is necessary to focus on assessing the relationship between financial coverage and health-care use toward a proper understanding of effective access to health care. Furthermore, this approach is able to shed light on the specific effects of public policies directed at increasing effective access to health services; it allows for operationalization of the measurement of a central element of universal health-care systems.

The World Health Organization has defined universal health-care coverage as the opportunity for any individual to use curative health-care services, preventative services, or health-promotion services that are of sufficient quality to be effective and that do not expose the user to financial hardship [6,7]. The Royal Society of Medicine has stated that the concept of access to health-care services requires complex analysis. For measurement purposes, access to health-care services can be approximated by quantifying the number of people who require medical care compared with the number that are actually admitted into the health-care system. The concept of access to health care needs to be addressed in at least four dimensions [8]:

• Availability of services.

• Ability to provide equitable health-care services. Access to services can be evaluated as a standardized relationship between health-care requirements and usage of services.

• The existence of personal, financial, organizational, social, and cultural barriers to access to health-care services.

• Health-care outcomes.

In Mexico, scholars have searched for an operational indicator of universal health-care coverage that reflects not only financial protection (understood as prepayments for health care to avoid out-of pocket payments) but also the opportunity of use (in terms of adequate quantity and quality), as defined by the system’s different access options and its responsiveness. The lack of any of these three dimensions (use of needed services, service quality, and financial protection) implies an absence of effective access, and such access is what ultimately ensures universal coverage [9]. Lack of effective access owing to the absence of financial protection is the easiest dimension to measure: if an individual does not have health insurance, he or she lacks sufficient access. Measuring the other two dimensions is more complex and requires additional assumptions. Specifically, the use of services and service quality are not independent variables: they are highly correlated.

In the case of Mexico, financial protection has been channeled through two large public mechanisms: social security and the SPSS. Whereas social security includes institutions that both finance and supply health-care services, the SPSS (coordinated by the National Commission) primarily finances services offered by other entities, such as state health services and federal hospitals. These financing mechanisms rely on a public network of health-care suppliers, which are allocated resources according to the number of individuals for whom they provide services. Thus, the aim under Mexico’s financing strategy is that universal coverage is achieved by offering the option to access sufficient services of sufficient quality, financed by either social security or the SPSS, the vast majority of which are public services.

As a consequence of this situation, an access problem may be indicated when the population insured through either social security or the SPSS makes out-of-pocket payments for health-care services. It is assumed that in a situation with opportune (adequately located and timely) and efficient health-care services financed by social security or the SPSS, individuals will elect to use these services whenever the need arises. Conversely, when faced with problems of service availability or quality, individuals will be willing to make out-of-pocket payments for health-care services outside the network.

We propose therefore that any individual with public health insurance who makes use of private services through direct payments (excluding private prepayments) does not have effective access to health-care services. This assumption implies that once an individual (or household) decides on the need to use health services, the selection of provider depends on the intrinsic characteristics of available suppliers, declared price, opportuneness (including location and time) and quality (perceived). If opportunity and quality are similar among providers, it is expected that price will determine the decision. Individuals with insurance (financial protection) will therefore always opt for providers within these financial schemes *ceteris paribus*. However, if individuals perceive challenges in opportuneness or quality and have available resources, they may opt to pay out-of-pocket for other services that may be more convenient or perceived as more effective.

An additional issue related to individual preferences (not covered by elements of opportuneness and perceived quality) could be the perception of private health-care provision as a luxury good; in this way, an individual—even when having insurance—may prefer expensive private services [10]. Nevertheless, this is not assumed to be highly prevalent.

The objective of the present study is to propose and estimate an indicator for effective access to health-care services in Mexico. Such an indicator needs to permit measurement of a task’s magnitude and be effective in monitoring progress. The proposed indicator was assessed using data collected by the 2006 and 2012 National Health and Nutrition Survey (ENSANUT—Encuesta Nacional de Salud y Nutrición [in Spanish]).

## Methods

As explained in the Introduction, effective access is proposed as an operational measure of universal coverage and integrates its three dimensions: use of needed services, service quality, and financial protection.

### Data

The data used in this analysis were extracted from the 2006 and 2012 ENSANUT surveys; specifically, they were taken from the survey instruments that included the variables of household financial protection and user access to health-care services, the general household instrument, and the health-services user instrument. A more detailed description of the design and scope of both the 2006 and 2012 ENSANUT surveys is reported elsewhere [11,12]. The ENSANUT sampling is representative of the national population in Mexico. The survey also includes details of state representation and representation by urban and rural strata at the national level. All ENSANUT data are openly available at http://ensanut.insp.mx.

The ENSANUT 2006 collected data from 47,152 households distributed among the 32 federal states in Mexico; that number represented 97% of selected households. In the survey, data were collected from an appropriate informant (a household member aged 18 years or older possessing information about all members) about general household characteristics and on its members’ sociodemographic details, including health insurance and utilization of hospital services. Data were also obtained from the household informant about recent utilization of ambulatory health services by all household members. In those households where at least one member was reported to have used ambulatory health services in the previous 2 weeks, a user was selected for direct interview. From all the surveyed households, use of recent health services was indicated in 12,928, and data were collected from 12,860 users (99% of those identified).

The procedures and structure for the ENSANUT 2012 survey were similar to those in 2006. Data were collected from 50,528 households—92% of those selected. Among these, 14,885 individuals were selected for the recent health user’s instrument, and data were collected from 14,104 (95%).

There was one important difference in the health services user instrument between the 2006 and 2012 surveys: in the 2006 survey, the use of preventive services was included in identification of use, though this was not the case in the 2012 survey. However, in 2006, 91% of users attended health services for non-preventive interventions, and the analysis was restricted to this group to ensure comparability.

Datasets for both surveys included expansion factors (weights), which allowed the estimates to be generalized for the Mexican population. Expansion factors for the health-care service user dataset allowed expansion to those individuals that utilized the services in the 2 weeks before the households were visited. In this analysis, it was assumed that users’ behavior represented utilization for the entire population. From the utilization rates (5.8% in 2006, 7.7% in 2012), it could be assumed that all individuals used health services at least once a year; since the time of the survey was exogenous to households, those interviewed could be seen as a random sample of all individuals. Therefore, expansion factors from the users were adjusted to the total country population.

### Variables

Financial protection is defined as an affiliation to any relatively comprehensive insurance program, which means a program that includes services ranging from basic outpatient services to inpatient hospital care. In Mexico, such a program includes the population covered by social security and the SPSS. Although interventions in the two schemes are different, both are considered comprehensive systems. In both the 2006 and 2012 surveys, affiliation for each household member was reported by the household informant, who answered for all individuals the question “Is [NAME] affiliated or registered to health services from … ?”; a list of institutions and insurance schemes was read for the last item, with none being the final option.

The use of private outpatient services in the financially protected population has been identified as a proxy measure of the limitations of public services; it is influenced by any barrier to access and emphasizes the perception of inadequate quality or efficiency and insufficient times or locations [13,14]. The use of private health services by those with public financial protection was identified using the health-services user instrument. Among those that attended ambulatory health services for non-preventive services, both financial protection status (from the household informant report) and provider were identified. The provider was reported by the interviewee in answer to the following item in the questionnaire: “What was the institutional affiliation of the person that provided health care to you?” The interviewer indicated the reported institution in a comprehensive list of public providers, which also included private ones as an option.

As the first step in this measurement, the population with financial protection that utilized private services was identified among those having declared a health problem and having used health-care services. With the aim of calculating the percentage of the total population as the indicator of effective access, this group was used as the numerator and the total population as the denominator; we thus calculated the percentage of the population with financial protection and that had used private services when seeking care for a health problem.

Additionally, the data were stratified according to sex and socioeconomic level. Socioeconomic level was estimated in both the 2006 and 2012 surveys using an imputation strategy, which is described in detail elsewhere [15]. The strategy uses data on sociodemographic characteristics of the head of the household, household composition, and housing conditions from the National Income and Expenditures Survey. The latter is a nationally representative survey that is implemented every 2 years in Mexico to predict per capita income quintiles for households; it uses the coefficients to impute the quintile for the ENSANUT households. All members of a household were considered to be on the same socioeconomic level.

The use of hospitalization services was identified by the household informant. For each household member, the informant answered the question, “Was [NAME] hospitalized during the past year?”; “yes” and “no” were the possible answers.

### Analysis

The 2006 and 2012 ENSANUT survey data were used to estimate the indicator of effective access to ambulatory services, defined as:

Effective access = 100% – (% population without financial protection) – (% population with financial protection * % population with financial protection that used private health care because of access barriers to public services). As noted above, financial protection was identified from the household instrument, and the health provider was reported during the user interviews. The data were consolidated to estimate the effective access in 2006 and 2012. Thus, the proportion of the population with effective access and the 95% confidence intervals were estimated.

Year and federal state comparisons were performed by comparing the 95% confidence intervals of the estimates to establish whether those values could be considered different with 95% confidence. Finally, an indicator of effective access to hospitalization services was estimated using data from those individuals reported to have been hospitalized in the previous year to the visit. Since this was a small proportion of the population (3.5% in 2006, 3.8% in 2012), estimations were made only for this group; i.e., it was assumed that their behavior did not reflect that of the entire population.

## Results

Tables 1 and 2 show the 2006 and 2012 estimates for the percentages of the population without financial protection and without effective use of health-care services for the total population. The results are stratified by sex and socioeconomic quintile. The tables also show the resulting estimates for the population with effective access to health-care services for the same strata.

**Table 1:** Percentages (95% confidence intervals) of the Mexican population without financial protection, without effective use of health-care services, and with effective access to such services in 2006

	**Population percentage without financial protection**	**Population percentage without effective use**	**Population percentage with effective access**
**Total**	55.71	10.50	**33.79**
	(54.61 - 56.81)	(9.61 - 11.39)	**(32.15 - 35.43)**
**Men**	56.60	11.10	**32.30**
	(53.92 - 59.27)	(9.74 - 12.46)	**(30.03 - 34.58)**
**Women**	55.12	10.10	**34.78**
	(52.82 - 57.42)	(9.04 - 11.15)	**(32.74 - 36.83)**
**Quintile 1**	63.15	6.07	**30.78**
	(60.28 - 66.03)	(5.14 - 7.00)	**(28.16 - 33.41)**
**Quintile 2**	60.22	10.12	**29.66**
	(56.90 - 63.54)	(8.32 - 11.92)	**(27.00 - 32.33)**
**Quintile 3**	48.38	13.28	**38.33**
	(44.43 - 52.34)	(11.14 - 15.43)	**(34.88 - 41.78)**
**Quintile 4**	43.56	16.03	**40.40**
	(39.24 - 47.89)	(13.34 - 18.72)	**(36.48 - 44.32)**
**Quintile 5**	40.63	20.07	**39.30**
	(34.38 - 46.87)	(15.47 - 24.67)	**(34.05 - 44.56)**
**Social Security**		25.16	**74.84**
		(23.21 - 27.10)	**(72.90 - 76.79)**
***Seguro Popular***		19.03	**80.97**
		(15.96 - 22.11)	**(77.89 - 84.04)**

Source: own estimates based on the 2006 ENSANUT.

**Table 2:** Percentages (95% confidence intervals) of the Mexican population without financial protection, without effective use of health-care services, and with effective access to such services in 2012

	**Population percentage without financial protection**	**Population percentage without effective use**	**Population percentage with effective access**
**Total**	26.66	21.83	**51.51**
	(26.00 - 27.33)	(20.57 - 23.09)	**(50.02 - 53.00)**
**Men**	28.76	23.51	**47.89**
	(28.04 - 29.48)	(21.70 - 25.33)	**(45.70 - 50.07)**
**Women**	24.66	20.72	**53.90**
	(23.95 - 25.37)	(19.28 - 22.15)	**(52.10 - 55.70)**
**Quintile 1**	25.51	14.71	**62.86**
	(24.40 - 26.63)	(12.78 - 16.64)	**(59.87 - 65.86)**
**Quintile 2**	26.92	20.50	**53.46**
	(25.67 - 28.17)	(18.08 - 22.91)	**(50.15 - 56.77)**
**Quintile 3**	26.66	21.56	**52.90**
	(25.38 - 27.93)	(19.05 - 24.08)	**(49.71 - 56.09)**
**Quintile 4**	27.39	24.88	**45.99**
	(26.14 - 28.65)	(22.10 - 27.66)	**(43.20 - 48.78)**
**Quintile 5**	27.55	28.00	**41.56**
	(26.10 - 28.99)	(24.96 - 31.04)	**(38.41 - 44.71)**
**Social Security**		29.77	**70.23**
		(27.72 - 31.81)	**(68.19 - 72.28)**
***Seguro Popular***		29.76	**70.24**
		(27.47 - 32.06)	**(67.94 - 72.53)**

Source: own estimates based on the 2012 ENSANUT.

The results for the 2006 ENSANUT indicated that 55.71% of the population lacked financial protection for health care. Despite being financially protected, 10.50% of the total population opted for private ambulatory services. Therefore, in 2006, the Mexican population with effective access to health-care services was 33.79%.

Effective access was slightly higher for women than for men (34.78% vs. 32.30%), and the highest socioeconomic quintile had a higher proportion of the population with effective access than the lowest quintile (39.30% vs. 30.78%). In the population with financial protection, there was a larger proportion of individuals without effective use of health-care services among people with social security (25.16%) than those covered by Seguro Popular (19.03%). This may reflect the stricter financial constraints faced by people in the Seguro Popular (lower-income population), which limits their choices in terms of accessing private services.

The results for the 2012 ENSANUT survey show that 26.66% of the population lacked a financial protection scheme. In the population with financial protection, approximately one-third of those with public insurance used private services in their last outpatient consultation, representing 21.83% of the total population. The proportion of the total population with effective access to health-care services was estimated at 51.51%, and once again the percentage was higher for women than for men (53.90% vs. 47.89%). According to socioeconomic status, the proportion of the total population with effective access was highest for the lowest income quintile—62.86%—compared with the highest income quintile, in which 41.56% of the population had effective access. We can therefore conclude that for the total population (even adjusted by sex) and for the lowest three socioeconomic quintiles, effective access increased from 2006 to 2012 since their confidence intervals did not overlap. However, the confidence intervals for the two highest socioeconomic quintiles did overlap, which suggests that the changes between 2006 and 2012 for those quintiles may not be significant. In other words, effective access for the two highest socioeconomic quintiles did not significantly change between 2006 and 2012.

A noteworthy and important result is that while the percentage of the population lacking financial protection showed a downward trend between 2006 and 2012, the proportion of users with problems in the effective use of health-care services also changed significantly during that period (10.50% vs. 21.83%). Though further analysis is needed to explain this increment, it is partially explained by the increase in the size of the potential population for this indicator: since this indicator is estimated from individuals with financial protection, the rise in this group also increased the size of the population for the estimation of effective use. As a percentage of the population with financial protection, individuals without effective use amounted to 23.73% in 2006 and 29.77% in 2012, i.e., a 25% increase. By 2012, there was no difference in effective use of or access to health-care services between people covered by social security and those covered by Seguro Popular.

**Figure 1:**
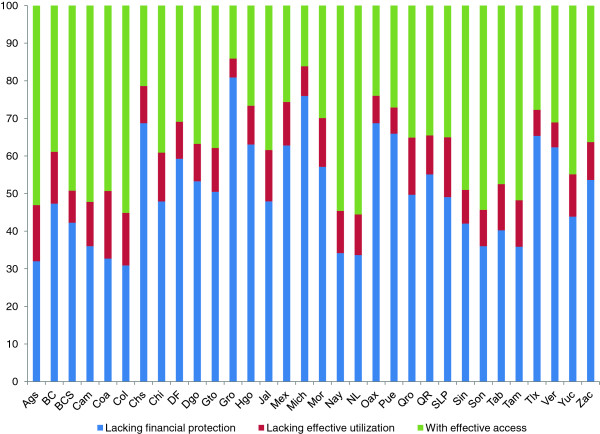
Percentages of the population without financial protection, without effective use of health-care services, and with effective access to such services by federal state in Mexico in 2006.

**Figure 2:**
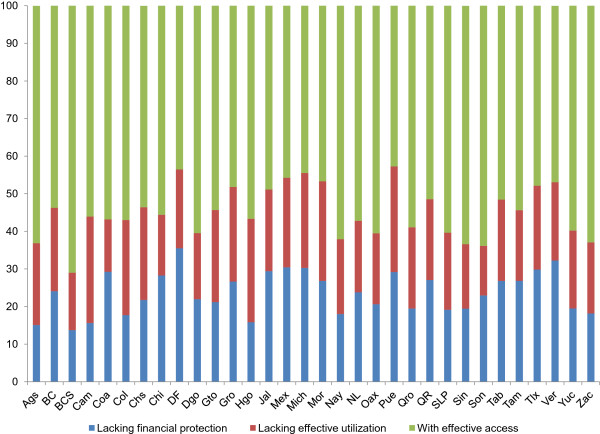
Percentages of the population without financial protection, without effective use of health-care services, and with effective access to such services by federal state in Mexico in 2012.

With respect to regional variations, the proportions of the population without financial protection and without effective use of health-care services by federal state are shown in Figures 1 and 2 for 2006 and 2012, respectively. The results show ample heterogeneity among the states for both years, though the heterogeneity was reduced in 2012. In addition, the increase in financial protection occurred concurrently with an increase in problems with the effective use of the services. Comparisons among the states were performed at the 95% confidence intervals for the estimated indicator for effective access (i.e., the difference between each bar in Figures 1 and 2 and 100%). There was considerable overlapping, which could be attributable to the sample sizes. Even so, when comparing, for example, the states with the highest and lowest proportions of effective access, the intervals did not overlap, which indicates that effective access was different with 95% confidence.

Finally, the results of the indicator for effective access to hospitalization appear in Table 3. Effective access to hospital services was 49.78% for individuals that required hospitalization in 2006. A key element related to the larger percentage for this indicator compared with effective access to ambulatory services is financial protection coverage. Among individuals that required in-patient services, 42.69% lacked financial protection, compared with 55.71% for people that required ambulatory services. Effective access to hospitalization increased to 75.49% in 2012, or 70.43% when adjusted for those covered by Seguro Popular that required interventions not covered by SPSS and therefore were considered as being without effective use of services. For 2012, it is important to note that while lack of financial protection among individuals that required ambulatory services amounted to 26.66%, it was 14.03% for those that required in-patient services.

**Table 3:** Percentages (95% confidence intervals) of the Mexican population that required in-patient services without financial protection, without effective use of health-care services, and with effective access to in-patient services in 2006 and 2012

	**Population percentage without financial protection of those that required in-patient services**	**Population percentage without effective use of those that required in-patient services**	**Population percentage with effective access to in-patient services**
**2006**	42.69	7.52	**49.78**
	(40.56 - 44.83)	(6.56 - 8.49)	**(47.71 - 51.85)**
**2012**	14.03	10.48	**75.49**
	(12.83 - 15.23)	(9.45 - 11.52)	**(74.03 - 76.95)**
2012 adjusted*	14.03	18.68	**70.43**
	(12.83 - 15.23)	(17.37 - 20.00)	**(68.87 - 71.99)**

*Adjustment was made to exclude from effective use those with financial protection by the Seguro Popular that demanded in-patient services that were not covered by the SPSS. Household informant reported whether inpatient cost were covered or not by the SPSS.

## Discussion

The proposed indicator suggests that just over half of the Mexican population had effective access to health-care services in 2012 and that about 30% of individuals requiring in-patient services lacked effective access to such services. Therefore, the challenge to ensure universal effective access amounts to incorporating the remaining half of the Mexican population. Considering the country’s estimated population in 2012, approximately 57 million people presently do not have effective access to health-care services.

In terms of the relative weight of the two factors considered in the present study, just over half (54.3%) of the lack of effective access was a result of the absence of financial protection. The remainder (45.7%) was due to what are defined in this study as limitations or barriers to public health-care services. These limitations or barriers may be because of shortcomings in quality or lack of geographic availability or opportune access when facing a health-care need.

It is important to note that the measure of effective access may be an underestimation of the challenge faced by the Mexican health system. As indicated above, effective access seems to be higher among individuals in the lower socioeconomic quintiles, but this may only reflect a lack of resources to opt for other services, i.e., the proposed indicator does not capture *intention* to use other services but the actual use of such services.

Regarding the indicator related to effective access to in-patient services, a key element among the results was that individuals who used hospital services were more likely to have financial protection. Since there is no evidence that those without financial protection are less likely to need in-patient services, it could be that this partly reflects a financial barrier.

The proposed indicator allowed us to comparatively examine the changes between 2006 and 2012. In addition to finding a significant increase in effective access (33.79% vs. 51.51%) during this period, the indicator also permitted us to distinguish between the specific contributions of the two considered elements. On one hand, the rise in effective access was associated with a substantial increase in financial protection (44.29% vs. 73.40%); this was concurrent with a reduction in the opportunity for effective health-care service use, as evident in the increased percentage of the population lacking effective use (10.50% vs. 21.83%).

Comparisons among the federal states permitted the identification of significant differences between the extremes, that is, between states with the lowest & highest levels of effective access. The results suggest that the indicator can be used for regional comparisons to categorize which characteristics are associated with the best (and worst) results.

It is important to note that with the variable of private health-care service use by the financially protected population, the use pattern among individuals that presented health problems in the 2 weeks before surveys was assumed to be similar to that of the total population. In other words, the observed pattern was independent of the characteristics of the individuals who reported service use in the study period: the pattern was similar regardless of the period and the group that presented health problems. Nevertheless, this assumption does not appear to be very restrictive.

Ensuring effective access to health-care services is the means toward achieving universal health-care coverage. Mexico has made very significant progress in this direction, particularly in terms of financial protection, yet half of the population still lacks effective access.

Analysis of the changes reflected in the indicators for 2006 and 2012 reveals a relative empowerment of women in terms of effective access to health care. The proportion increased from 34.78% to 53.90% for all women in the country. This result shows important progress in what has been highlighted as a priority by the health-care services. Similarly, whereas in 2006, the lowest socioeconomic quintile had significantly lower effective access than the highest quintile (quintile 1, 30.78% vs. quintile 5, 39.30%), the situation reversed by 2012. In that year, the proportion of effective access for the highest quintile was maintained, while the proportion for the lowest quintile significantly increased (quintile 1, 62.86% vs. quintile 5, 41.56%).

It is most likely that the population in higher socioeconomic levels is less concerned with access to private services and associated out-of-pocket expenses. However, between 37.14% and 47.10% of the population in the three lowest socioeconomic quintiles were still estimated to be in this situation (i.e., the remaining population in those quintiles without effective access). This result represents an unequivocal call to reinforce efforts toward improving access to financial protection and for the availability, suitability, and quality of health-care services.

## Conclusion

This study shows that it is possible to evaluate the progress achieved through public policies focused on establishing a universal national health system with effective access to health-care services. The proposed indicator allowed us to obtain a reference for the degree of effective access and observe the outcomes of particular policies and programs in the total population as well as different subgroups. In addition, the indicator enabled us to follow the progression of effective access over time and with specified periodicity.

## Competing interests

The authors declare that they have no competing interests.

## Authors’ contributions

JPG led the design and interpretation of the analysis and drafted the manuscript. SGS contributed to the interpretation and to the drafting of the manuscript. GFD and MHA provided advice on the interpretation and reviewed the manuscript. All authors read and approved the final manuscript.

## Pre-publication history

The pre-publication history for this paper can be accessed here:

http://www.biomedcentral.com/1472-6963/14/186/prepub

## References

[B1] Frenk J Gonzalez-Pier E Gómez-Dantes O Lezana MA Knaul FM Comprehensive reform to improve health system performance in Mexico Lancet 2006 14 9546 1524 1534 10.1016/S0140-6736(06)69564-0 17071286

[B2] Frenk J Bridging the divide: global lessons from evidence-based health policy in Mexico Lancet 2006 14 9539 954 961 10.1016/S0140-6736(06)69376-8 16962886

[B3] Secretaría-de-Salud Diagnóstico del financiamiento de la salud en México 2006 2 México: Secretaría de Salud

[B4] Narro RJ Moctezuma ND Orozco HL Hacia un nuevo modelo de seguridad social Economía UNAM 2010 14 20 7 33

[B5] Frenk J The Global Health System: Strengthening National Health Systems as the Next Step for Global Progress PLoS Med 2010 14 1 e1000089 10.1371/journal.pmed.1000089 20069038 PMC2797599

[B6] WHO World Health Report 2010. Health systems financing. Path to universal coverage 2010 Geneva: World Health Organization 10.2471/BLT.10.078741 PMC2878164 20539847

[B7] Frenk J de Ferranti D Universal health coverage: good health, good economics Lancet 2012 14 9845 862 864 10.1016/S0140-6736(12)61341-5 22959372

[B8] Gulliford M Figueroa-Muñoz J Morgan M Hughes D Gibson B Beech R Hudson M What does “access to health care” mean? J Health Serv Res Policy 2002 14 3 186 188 10.1258/135581902760082517 12171751

[B9] ILO Social health protection: an ILO strategy towards universal access to health care 2008 Geneva: International Labour Organization

[B10] Makinen M Waters H Rauch M Almagambetova N Bitran R Gilson L McIntyre D Pannarunothai S Prieto AL Ubilla G Ram S Inequalities in health care use and expenditures: empirical data from eight developing countries and countries in transition Bull World Health Organ 2000 14 1 55 65 10686733 PMC2560608

[B11] Romero-Martínez M Shamah-Levy T Franco-Núñez A Villalpando S Cuevas-Nasu L Gutiérrez JP Rivera-Dommarco J Encuesta Nacional de Salud y Nutrición 2012: diseño y cobertura Salud Publica Mex 2012 14 S2 332 340 24626712

[B12] Olaiz-Fernández G Rivera-Dommarco J Shamah-Levy T Rojas R Villalpando-Hernández S Hernández-Avila M Sepúlveda-Amor J Encuesta Nacional de Salud y Nutrición 2006 2006 Instituto Nacional de Salud Pública: Cuernavaca, México

[B13] Levesque J-F Harris M Russell G Patient-centred access to health care: conceptualising access at the interface of health systems and populations Int J Equity Health 2013 14 1 18 10.1186/1475-9276-12-18 23496984 PMC3610159

[B14] Secretaría-de-Salud Observatorio de los Servicios de Atención Primaria 2012 Dirección General de Evaluación del Desempeño 2013 México: Secretaría de Salud

[B15] Gutiérrez JP Clasificación por niveles socioeconómicos de los hogares entrevistados para la Encuesta Nacional de Salud y Nutrición 2012 Salud Publica Mex 2012 14 S2 341 346

